# Effect of a baby‐friendly workplace support intervention on exclusive breastfeeding in Kenya

**DOI:** 10.1111/mcn.13191

**Published:** 2021-04-08

**Authors:** Elizabeth W. Kimani‐Murage, Calistus Wilunda, Teresia Njoki Macharia, Eva Watiri Kamande, Peter Muriuki Gatheru, Tadesse Zerfu, Hermann Pythagore Pierre Donfouet, Laura Kiige, Susan Jabando, Lynette Aoko Dinga, Betty Samburu, Richard Lilford, Paula Griffiths, Debra Jackson, France Begin, Grainne Moloney

**Affiliations:** ^1^ Maternal and Child Wellbeing Unit African Population and Health Research Center Nairobi Kenya; ^2^ Epidemiology and Prevention Group, National Cancer Center Tokyo Japan; ^3^ United Nations Children's Fund (UNICEF) N'djamena Chad; ^4^ United Nations Children's Fund (UNICEF) Nairobi Kenya; ^5^ Department of Food Science and Technology Jomo Kenyatta University of Agriculture and Technology Juja Kenya; ^6^ Warwick Medical School University of Warwick Coventry UK; ^7^ School of Sport, Exercise and Health Sciences Loughborough University Loughborough UK; ^8^ School of Clinical Medicine University of the Witwatersrand Johannesburg South Africa; ^9^ United Nations Children's Fund (UNICEF) Headquarters New York USA; ^10^ MARCH Centre London School of Hygiene and Tropical Medicine London UK; ^11^ School of Public Health University of the Western Cape Bellville South Africa

**Keywords:** baby‐friendly workplace, breastfeeding support, infant feeding behaviour, mother‐friendly workplace, propensity score weighting

## Abstract

Exclusive breastfeeding (EBF) during the first 6 months of life is crucial for optimizing child growth, development and survival, as well as the mother's wellbeing. Mother's employment may hinder optimal breastfeeding, especially in the first 6 months. We assessed the effectiveness of a baby‐friendly workplace support intervention on EBF in Kenya. This pre‐post intervention study was conducted between 2016 and 2018 on an agricultural farm in Kericho County. The intervention targeted pregnant/breastfeeding women residing on the farm and consisted of workplace support policies and programme interventions including providing breastfeeding flexi‐time and breaks for breastfeeding mothers; day‐care centres (crèches) for babies near the workplace and lactation centres with facilities for breast milk expression and storage at the crèches; creating awareness on available workplace support for breastfeeding policies; and home‐based nutritional counselling for pregnant and breastfeeding women. EBF was measured through 24‐h recall. The effect of the intervention on EBF was estimated using propensity score weighting. The study included 270 and 146 mother–child dyads in the nontreated (preintervention) group and treated (intervention) group, respectively. The prevalence of EBF was higher in the treated group (80.8%) than in the nontreated group (20.2%); corresponding to a fourfold increased probability of EBF [risk ratio (RR) 3.90; 95% confidence interval (CI) 2.95–5.15]. The effect of the intervention was stronger among children aged 3–5 months (RR 8.13; 95% CI 4.23–15.64) than among those aged <3 months (RR 2.79; 95% CI 2.09–3.73). The baby‐friendly workplace support intervention promoted EBF especially beyond 3 months in this setting.

**Key messages**

- Mother's employment may hinder optimal breastfeeding especially in the first 6 months.
- In this study, the baby‐friendly workplace support intervention promoted EBF and were particularly supportive in increasing EBF likelihood beyond 3 months, which is the age in Kenya beyond which support for maternity leave ceases for those working in the formal sector.
- Maintaining EBF while working is more likely when employers provide the support that women need to do so, thus there is a need for policies and programmes at workplaces to support women to combine work with breastfeeding.

## 1 INTRODUCTION

Child mortality remains an overarching global development challenge (United Nations, 2015). Although the global under‐five mortality rate fell from 93 deaths per 1,000 live births (12.6 million deaths) in 1990 to 39 deaths per 1,000 live births (5.3 million deaths) in 2018, this average reduction masks stark disparities across regions and countries. For instance, sub‐Saharan Africa remains the region with the highest under‐five mortality rate in the world, with the risk of death before the fifth birthday being 15 times higher than that in high‐income countries (United Nations Inter‐agency Group for Child Mortality Estimation, 2019). Infants accounted for about 75% of under‐five deaths in 2018, with mortality risk being highest during the neonatal period (United Nations Inter‐agency Group for Child Mortality Estimation, 2019). This pattern also applies to Kenya where the under‐five mortality rate was 52 deaths per 1,000 live births according to a 2014 national survey (Kenya National Bureau of Statistics et al., 2015).

To reduce child mortality, the World Health Organization (WHO) and United Nations Children's Fund (UNICEF) recommend, among other actions, exclusive breastfeeding (EBF) during the first 6 months of life (World Health Organization, 2017). EBF reduces the risk of infant morbidity, hospitalization and mortality (Lamberti et al., 2011, 2013; Sankar et al., 2015). The benefits of breastfeeding to the child extend well beyond the breastfeeding period and include a lower risk of obesity (Horta et al., 2015b), asthma (Lodge et al., 2015), malocclusion (Peres et al., 2015) and an increased intelligence quotient (Horta et al., 2015a). Moreover, breastfeeding mothers have a lower risk of breast cancer, ovarian cancer, type II diabetes and postpartum depression (Chowdhury et al., 2015). However, despite these proven benefits of breastfeeding, globally, only 41% of infants younger than 6 months are exclusively breastfed (UNICEF & WHO, 2019). In Kenya, the prevalence of EBF among children aged 0–6 months was slightly higher at 61% in 2014 (Kenya National Bureau of Statistics et al., 2015).

Mother's employment may hinder optimal breastfeeding (Guendelman et al., 2009; Skafida, 2012). This may be due to lack of adequate maternity leave (Navarro‐Rosenblatt & Garmendia, 2018), breastfeeding breaks, adequate facilities for expressing and storing milk, resources that promote breastfeeding, and support from employers and co‐workers of mothers (Tsai, 2013). As a result, the rate of breastfeeding among employed mothers rapidly decreases after resuming work (Chuang et al., 2010).

In Kenya, 64% of women aged 15 years and above are in the workforce, most of them in the agricultural sector (World Bank, 2019). The pressure to work long hours to make sufficient income, inability to maintain a work‐life balance and lack of support at the workplace are some of the factors that diminish breastfeeding rates among working mothers (Kimani‐Murage et al., 2015; Philips Africa Innovation Hub, 2015). Moreover, the 3‐month paid maternity leave provided in the current Kenya Employment Act (National Council for Law Reporting, 2012) is insufficient to support EBF for the first 6 months, as 52% of mothers resume work within 3 months after birth—driven by the fear of losing their jobs and the need to provide for their families (Philips Africa Innovation Hub, 2015). This affects optimal breastfeeding, highlighting the need to promote a breastfeeding‐friendly workplace for the employed mothers (Mills, 2009).

The Baby‐Friendly Workplace Initiative (also called Mother‐Friendly Workplace Initiative) was launched in 1993 to promote combining women's work and breastfeeding (World Alliance for Breastfeeding Action, 1993). The objective was to complement the baby‐friendly hospital initiative and extend baby‐friendliness beyond the hospital walls and into women's working environments. The initiative consists of three concepts: time, space/proximity and support (Yimyam & Hanpa, 2014). Time includes providing paid maternity leave, flexible working hours and breastfeeding breaks. Space/proximity includes providing infant/child care at or near the workplace, facilities for expressing and storing breastmilk, and environmental safety. Support involves informing women about maternity benefits, among others. In 2019, UNICEF issued an evidence brief on family‐friendly policies that workplaces can implement to ensure they are supporting their working parents and caregivers (UNICEF, 2019). Workplace support for breastfeeding is essential for continued breastfeeding in Kenya's Agricultural sector, which employs about 60% of women (World Bank, 2020). Poor working conditions and access to health services for workers in the agricultural sector have been documented (Gitonga, 2009). Women who work in the agricultural export sector may have limited exposure and accessibility to community‐based health education programmes (Andrieu et al., 2014). Studies from other countries show that workplace support for breastfeeding interventions may promote appropriate infant and young child feeding practices (Kim et al., 2019). However, there is little evidence, especially from intervention studies, to support this premise in low‐income settings such as Kenya. Thus, we aimed to assess the effectiveness of a baby‐friendly workplace support intervention in promoting EBF in one of the largest agricultural estates in Kenya.

## 2 METHODS

### 2.1 Setting

The study was conducted in one of the large‐scale agricultural farms in Kericho County, in the highlands west of the Kenyan rift valley. The county, which is home to some of the largest agricultural estates in Kenya, covers an area of 2,111 km^2^ and a population of 739,872 of which 44% are aged 0–14 years. The study site covers over 8,700 ha and has a population of over 80,000 people in 112 villages, accounting for over 90% of the population living within the agricultural plantation. There are close to 16,000 employees, a third being women. The majority of the employees are seasonal workers, working on the farms, whereas the rest are permanent employees working within the factories, offices and as security personnel. There is an organized employer‐supported health care system, which includes a major (Level 4) hospital, four health centres (Level 3), and 23 dispensaries (Level 2), and a comprehensive HIV/AIDs programme. The agricultural estate also has other social facilities including staff houses, social halls, schools (20 primary schools, 8 secondary schools and 53 early childhood development centres), clean water supply and electricity. The plantation has peer educators who work as volunteers on health and social matters.

### 2.2 Study design and population

The study employed an outcome evaluation as well as an implementation research study design in line with the WHO's Alliance for Health Policy and Systems Research (WHO/AHPSR) implementation research guide (Peters, Tran, & Adam, 2013) and the 2010 Quality Standards for Development of Evaluation by the Organization for Economic Co‐operation and Development/Development Assistance Committee [Organisation for Economic Co‐operation and Development (OECD), 2010]. The effectiveness‐implementation hybrid trials combined elements of implementation research and effectiveness to assess both the implementation strategy and the effectiveness of the initiative (Peters, Adam, et al., 2013). The participatory action research included innovative participatory methods such as photovoice and participatory videos, which encouraged the involvement of the beneficiaries/communities and co‐ownership of the initiatives to enhance transparency, accountability and capacity building of beneficiaries/community members. A community readiness assessment and other formative assessments were done at the beginning of the study. The information, collected through qualitative approaches and participatory methodologies, was used to tailor the intervention to the context at the intervention development stage and to adapt the intervention during the implementation. More details on this can be obtained from the published protocol paper (Kimani‐Murage et al., 2021).

The current paper focuses on the evaluation of the effect of the intervention on EBF. The evaluation employed a quasi‐experimental design, involving a pre‐post intervention design. The postintervention assessment was conducted after about 12 months of implementing the intervention. This evaluation design was deemed to be the most feasible evaluation design given that the intervention was designed to cover the entire study setting.

The study focused on female employees (permanent and seasonal), specifically mothers with infants (aged 0–12 months), and the infants themselves. Because this paper focuses on EBF for the first 6 months of life, we used data from mothers of children younger than 6 months.

### 2.3 Intervention

A preimplementation formative assessment to assess community readiness for the intervention (Center for Community Health and Development, 1994) and to engage the community and collect data necessary to tailor the intervention to the context in which it was applied was conducted before the implementation of the intervention (Kimani‐Murage et al., 2021). The formative assessment was conducted between September and November 2016. This was followed by a period of development of the intervention between December 2016 and April 2017. The intervention was then implemented for 12 months (from May 2017 to April 2018) (Kimani‐Murage et al., 2021).

The formative assessment revealed that the employing company had policies to support breastfeeding mothers. These included a 3‐month paid maternity leave for full‐time female workers, breastfeeding breaks, peer counsellors and flexible working hours. Eighty percent of the mothers were aware of these policies; however, several factors hampered their implementation. These included poor adherence to the policies by either the line managers or the mothers, long distances between the place of work and home (where the infants were) which hindered the utilization of the breastfeeding breaks, a lack of understanding of the importance of EBF by the managers, the mother and other employees, competing priorities—although some mothers desired to adhere to the policies they were forced to forgo breastfeeding to meet their minimum daily targets, and the volunteer peer‐educators were not empowered to educate the mothers on breastfeeding and combine it with work.

The intervention, which targeted all women living on the agricultural farm regardless of their employment status, consisted of advocacy, technical and collaborative financial support to the agricultural farm management to update and implement workplace support policies and programme interventions including providing paid breastfeeding breaks for breastfeeding mothers; establishing day‐care centres (crèches) for babies near the workplace where working mothers could access their babies for breastfeeding easily and lactation centres with facilities for breast milk expression and storage at the crèches; and creating awareness on both the value and the availability of workplace support for breastfeeding policies. There was also home‐based nutritional counselling through monthly visits for pregnant and breastfeeding women residing within the agricultural farm and nutrition education to other farmworkers to support breastfeeding. A detailed communication strategy was developed to provide a road map for behaviour change. The communication strategy was based on the socioecological model (Golden & Earp, 2012), which classified different spheres and key influencers to be targeted for behavioural interventions in maternal, newborn, and child health and nutrition (Figure S1).

The employing company refurbished available buildings into two daycare centres with dedicated rooms for expressing breastmilk, hired experienced nurses as caretakers to work in the centres, and provided equipment and supplies—including bottles for expressing breast milk, breast milk freezing and storage containers and fridges—for storing expressed breast milk. UNICEF provided early childhood development kits, television screens, digital versatile discs and videos on breastfeeding for training the mothers and other key influencers on good positioning, expression and storage of expressed milk. An existing workplace breastfeeding‐friendly policy by the employing company was revised based on the technical support provided and included implementation of the 3‐month paid maternity leave policy, allowing lactating mothers to take paid breastfeeding work breaks and flexibility in the time to report to work, and regular sensitization of women; their influencers (team members and supervisors) and management staff on the policy. Incentivized counsellors who were residents of the agricultural estate conducted home‐based nutritional counselling. The counsellors had received a 1‐week training, continuous support supervision, and mentorship and conducted house‐to‐house visits educating pregnant and lactating women and their partners on issues related to maternal, infant and young child nutrition (MIYCN) and childcare. To enhance their breastfeeding support, health workers, the supervisors of the counsellors and key welfare staff of the agricultural farm received an 8‐h/day, 6‐day training on MIYCN based on a standard curriculum developed by the Ministry of Health (2020). Health workers and incentivized counsellors created awareness about daycare centres, mobilized women to use the facilities and helped to form mother‐to‐mother support groups. A community‐based management structure called the community mother support group oversaw the day‐to‐day work of the incentivized counsellors, their supervisors, the health facility staff and the overall management of the programme. The employing company and UNICEF co‐financed the intervention.

### 2.4 Data collection

A preintervention survey was conducted between September and November 2016, whereas a postintervention survey was conducted between May and July 2018. The two study groups (i.e., for preintervention and postintervention survey) were independent of each other. All women with children aged less than 1 year and living in the plantation were recruited to assess their breastfeeding practices based on 24‐h recall using a questionnaire. The women were invited to participate in the study by filling a screening form administered by community health volunteers/peer educators or by health care workers during postnatal care. All eligible women agreed to participate. The questionnaire, which also collected socio‐demographic and economic data, was developed in English, translated to Swahili, programmed in Survey CTO software and uploaded in mobile phones for data collection. Trained research assistants collected data through face‐to‐face interviews. Supervision of the research assistants by field supervisors and members of the research team and regular review of the data were performed to ensure data quality.

### 2.5 Sample size and sampling procedures

A minimum sample of 600 women (i.e., 300 women in the preintervention group and 300 in the intervention group) was calculated assuming an increase in EBF from 17% in the preintervention group (hereafter referred to as the nontreated group) to 27% in the intervention group (hereafter referred to as the treated group), a two‐sided hypothesis test with a 5% significance level, a power of 80% and a non‐response rate of 10%. However, a comprehensive sampling was carried out by recruiting all consenting mothers with children under the age of 1 year. Accordingly, both at baseline and endline, consecutive mass recruitment of all mothers (employed permanently, casually or not working) who had children younger than 1 year and living in the agricultural plantation was followed.

### 2.6 Variables

The outcome variable was EBF, defined by WHO as consumption of only breastmilk and nothing else except oral rehydration fluids, drops or syrups in the past 24 h (Wold Health Organization, 2008). We considered, a priori, the following socio‐demographic variables as covariates based on their theoretical association with the intervention and/or breastfeeding: child's age in months (continuous) and sex, mother's age (1‐year interval); parity (1, 2, 3 and 4+); ethnicity (Kalenjin, Kisii and others); education (primary or less, secondary, and tertiary); religion (Christian and others); marital status (in a union and not in a union); and employment status (employed in the agricultural estate, employed elsewhere and unemployed).

### 2.7 Statistical analysis

Characteristics of the participants in the treated and nontreated groups were summarized using descriptive statistics. A propensity score, defined as the probability of being assigned to a treatment group given an individual's observed covariates (D'Agostino, 1998), was used to weight the sample and to ensure the covariates balanced across treatment groups. This approach is akin to applying survey weights in a sample survey. First, we generated propensity scores (using the ‘pscore’ command in Stata) by including the treatment variable and all the above covariates in the model. There was no evidence of covariate imbalance between the treated and nontreated groups within blocks of the propensity score. Next, we weighted the treatment groups by the propensity score based on the inverse probability of treatment weighting method using doubly robust estimation (Funk et al., 2011). Doubly robust estimation combines outcome regression and propensity score modelling to obtain an unbiased effect estimator (Funk et al., 2011). Each child in the intervention group received a weight equal to the inverse of the propensity score, whereas each comparison child received a weight equal to the inverse of one minus the propensity score (Garrido et al., 2014). The weighting variable was then included in a generalized linear model (Poisson regression with robust error variance) to assess the effect of the intervention on the outcome; expressed as risk ratio (RR) with 95% confidence interval (CI). We also assessed the effect of the intervention on the outcome in the usual way by using Poisson regression with robust error variance and adjusting for variables that showed some imbalance (at a conservative *P* < 0.2) between the treated and nontreated groups. Because this study included all women residing in the agricultural estate regardless of their employment status, we stratified the results by mother's employment status (i.e., employed in the estate or unemployed). Moreover, because the probability of EBF reduces with the child's age, we stratified the results by child's age (<3 months or 3–5 months). Because 47 participants in the nontreated group had missing outcome data, we compared the socio‐demographic characteristics of the participants with complete data and those with missing data and found no significant differences, apart from marital status (Table S1). We then performed sensitivity analysis to account for the missing data through multiple imputation using chained equations with 20 iterations. The imputation model included all the variables in Table 1 together with the treatment group. We then repeated the above analyses based on the imputed datasets and combined the estimates using Rubin's rules (Rubin, 1987). All analyses were conducted using Stata 15.1 and a two‐tailed *α* of 0.05.

**TABLE 1:** Characteristics of mothers and children in the non‐treated and treated groups

Characteristics	Non‐treated group (*N* = 270)	Treated group (*N* = 146)	*P* value
**Child's characteristics**			
Age, months			0.406
0.0–2.9	142 (52.6)	83 (56.9)	
3.0–5.9	128 (47.4)	63 (43.2)	
Sex			0.098
Male	156 (57.8)	72 (49.3)	
Female	114 (42.2)	74 (50.7)	
**Mother's characteristics**			
Age, years, mean ± SD	26.6 ± 6.3	27.6 ± 6.5	0.105
Parity			0.431
1	70 (25.9)	39 (26.7)	
2	70 (25.9)	28 (19.2)	
3	57 (21.1)	32 (21.9)	
4+	73 (27.0)	47 (32.2)	
Ethnic group			0.964
Kalenjin	135 (50.0)	75 (51.4)	
Kisii	69 (25.6)	36 (24.7)	
Other	66 (24.4)	35 (24.0)	
Education			0.752
Primary or less	138 (51.1)	69 (47.3)	
Secondary	95 (35.2)	55 (37.7)	
Tertiary	37 (13.7)	22 (15.1)	
Religion			0.019
Christian	256 (94.8)	145 (99.3)	
Other	14 (5.2)	1 (0.7)	
Marital status			0.029
Not in a union	61 (22.6)	20 (13.7)	
In a union	209 (77.4)	126 (86.3)	
Employment status			0.161
Employed in the agricultural estate	96 (35.6)	48 (32.9)	
Employed elsewhere	18 (6.7)	4 (2.7)	
Unemployed	156 (57.8)	94 (64.4)	

*Note*. Data are presented as *n* (%) except for mother's age, which is presented as mean ± SD. All *P* values are from Pearson's *χ* ^2^ tests, except for mother's age, which is from an independent samples *t*‐test.

### 2.8 Ethical considerations

Ethical approval for this study was granted by Amref Health Africa's Ethics and Scientific Review Committee (study protocol number: P231/2016). Written informed consent was obtained from all eligible participants. Participation in the study was voluntary and without any financial incentive.

## 3 RESULTS

This study included 270 and 146 mother–child dyads of children aged less than 6 months in the nontreated and treated groups, respectively (Table 1). There were no statistically significant differences between the study groups regarding participants' characteristics, except mother's religion and marital status. Mothers in the nontreated group were less likely to be in a union (*P* = 0.029) or Christians (*P* = 0.019) than those in the treated group.

**FIGURE 1:**
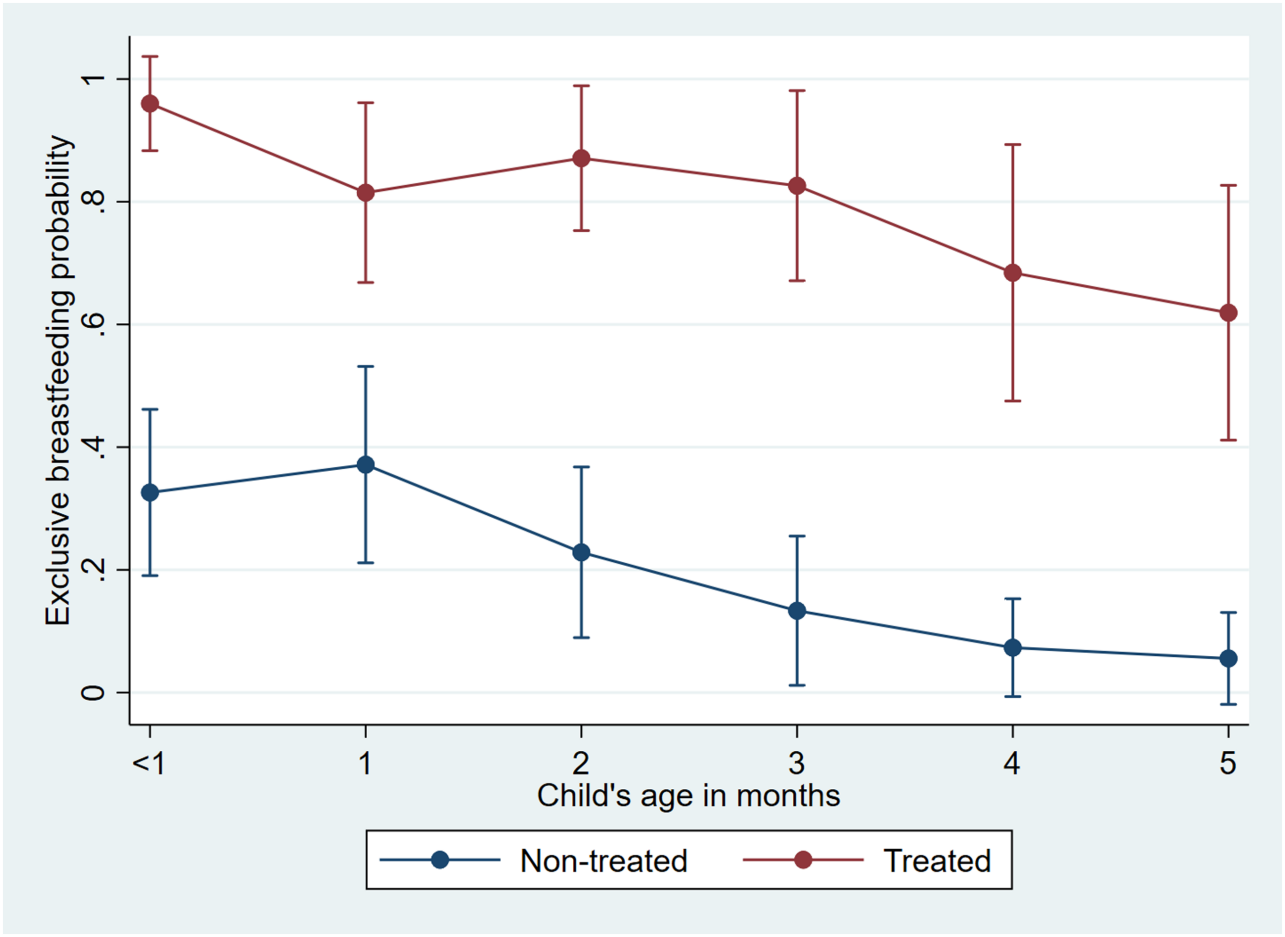
The probability of exclusive breastfeeding in treated and nontreated groups according to the child's age. The error bars represent 95% confidence intervals around the point estimates

The probability of reporting EBF was significantly higher in the treated group than in the nontreated group regardless of the child's age (Figure 1). In the treated group, the probability of reporting EBF was 96.0%, 82.6%, and 61.9% among children aged <1, 3 and 5 months, respectively. This corresponded to 32.6%, 13.3%, and 5.5% in the nontreated group. Table 2 shows the results of the effect of the intervention on EBF. The overall proportion of children younger than 6 months who were reported to be exclusively breastfed was higher in the treated group (80.8%) than in the nontreated group (20.2%). Propensity score weighted analysis showed an almost fourfold increased risk of reporting EBF in the treated group than in the nontreated group (RR 3.90; 95% CI 2.95–5.15). Similar results were obtained in the multivariable‐adjusted analysis. When the results were stratified by child's age (Table 2), a stronger effect of the intervention on reported EBF was observed among children aged 3–5 months (RR 8.13; 95% CI 4.23–15.64) than among those aged <3 months (RR 2.79; 95% CI 2.09–3.73). As Table 3 shows, the effect estimate was similar among children whose mothers were employed in the agricultural estate (RR 4.09; 95% CI 2.58–6.49) and among those whose mothers were unemployed (RR 3.63, 95% CI 2.53–5.19). The results of sensitivity analyses after multiple imputation to account for missing outcome data were similar to those of the main analysis where children with missing outcomes were excluded (Tables S2 and S3).

**TABLE 2:** Effect of the baby‐friendly workplace support intervention on exclusive breastfeeding

Study group	Exclusively breastfed	Unadjusted analysis	Propensity score weighted analysis	Multivariable adjusted analysis^a^
	*n* (%)	RR (95% CI)	RR (95% CI)	RR (95% CI)
**0.0–5.9 months**				
Nontreated (*N* = 223)	45 (20.2)	1	1	1
Treated (*N* = 146)	118 (80.8)	4.01 (3.05–5.26)	3.90 (2.95–5.15)	3.78 (2.85–5.01)
**0.0–2.9 months**				
Nontreated (*N* = 116)	36 (31.0)	1	1	1
Treated (*N* = 83)	73 (88.0)	2.83 (2.13–3.76)	2.79 (2.09–3.73)	2.67 (1.99–3.55)
**3.0–5.9 months**				
Nontreated (*N* = 107)	9 (8.4)	1	1	1
Treated (*N* = 63)	45 (71.4)	8.49 (4.45–16.21)	8.13 (4.23–15.64)	8.00 (4.25–15.03)

Abbreviations: CI, confidence interval; RR, risk ratio.

^a^ Adjusted for child's sex and mother's age, religion, marital status and employment status.

**TABLE 3:** Effect of the baby‐friendly workplace support intervention on exclusive breastfeeding stratified by mother's employment status

Study group	Mother employed in the agricultural estate (*N* = 126)			Mother unemployed (*N* = 225)		
	Exclusively breastfed	Propensity score weighted analysis	Multivariable adjusted analysis^a^	Exclusively breastfed	Propensity score weighted analysis	Multivariable adjusted analysis^a^
	*n* (%)	RR (95% CI)	RR (95% CI)	*n* (%)	RR (95% CI)	RR (95% CI)
Nontreated	16 (20.5)	1	1	27 (20.6)	1	1
Treated	40 (83.3)	4.09 (2.58–6.49)	3.81 (2.41–6.03)	74 (78.7)	3.63 (2.53–5.19)	3.50 (2.41–5.06)

Abbreviations: CI, confidence interval; RR, risk ratio.

^a^ Adjusted for child's sex and mother's age, marital status, religion and education.

## 4 DISCUSSION

The present study evaluated whether a baby‐friendly workplace support intervention promoted EBF among infants of mothers residing in a large agricultural estate in Kenya. We observed a fourfold increased probability of EBF, with a stronger effect among children aged 3–5 months than among those younger than 3 months.

Our results are generally consistent with those of a recent systematic review on the effectiveness of workplace lactation interventions on breastfeeding outcomes in the United States (Kim et al., 2019). The review found significant increases in EBF among mothers exposed to the workplace support interventions compared with those not exposed (Kim et al., 2019). However, differences in the study contexts, nature of the intervention packages, how EBF was measured, and the baseline EBF levels makes it difficult to directly compare our results with those of the studies included in the review by Kim et al. Given that breastfeeding among employed mothers rapidly decreases after returning to the workplace (Tsai, 2013), it was not surprising that the intervention had a stronger effect on EBF among older children. Thus, the intervention was more effective among mothers who were more likely to discontinue EBF because of having to return to work.

A systematic review found that providing a lactation space, breastfeeding breaks and comprehensive lactation support programmes were the three most common employer‐based programmes to support breastfeeding among working mothers (Dinour & Szaro, 2017). Although employers may perceive breastfeeding as a behaviour that may potentially hinder women's productivity, evidence shows that baby‐friendly workplace policy could improve employee's productivity, motivate mothers to return to work, reduce staff turnover and reduce absence associated with caring for a sick child (Cohen et al., 1995; Tuttle & Slavit, 2009). Thus, baby‐friendly workplace interventions are beneficial to mothers, infants and employers (UNICEF, 2019).

To the best of our knowledge, this is the first study to evaluate the effect of the baby‐friendly workplace support intervention on breastfeeding in sub‐Saharan Africa and findings confirm that interventions previously tested in high‐income settings can also benefit EBF in low‐income settings such as Kenya. We used propensity score weighting with doubly robust estimation to adjust for confounding and obtain unbiased effect estimates (Funk et al., 2011). Sensitivity analysis confirmed the robustness of our findings. The total cost of the intervention was US$ 87,973. Details of the economic evaluation of the intervention will be published in a separate paper. As of December 2020, the entire package of interventions except for daycare centres—which were closed because of the coronavirus disease‐19 pandemic—was ongoing, which underscores the sustainability of the intervention strategy. Nonetheless, this study has some limitations. First, there was staff layoff occasioned by the adoption of mechanical farming by the employing company during the intervention phase. This resulted in many women leaving the farm and reduced the number of women recruited during the postintervention survey, hence potentially reducing the power of the study. Despite this, a post hoc power analysis given the number of analysed participants and the increase in the prevalence of EBF from 20.2% at baseline to 80.8% at endline showed that the study was highly powered. Second, 47 children at baseline had missing outcome data. However, a comparison of the characteristics of the participants with complete data and those with missing data revealed no major systematic differences, and the results of sensitivity analyses after multiple imputation to account for the missing data were similar to those of complete‐case analysis, suggesting that the missing data were unlikely to have biased our results. Third, although we used a robust method to adjust for confounding, there may be other unmeasured confounders we could not account for. To account for such factors would have required a randomized controlled trial (RCT). Despite this, it is unlikely that confounding would explain the strong intervention effect observed in this study. Moreover, a Cochrane review on workplace interventions to support breastfeeding for women in employment found that no RCT or quasi‐RCT on this subject had been conducted (Abdulwadud & Snow, 2012), alluding to the practical challenges of evaluating such interventions through RCTs. Thus, evidence from quasi‐experimental studies, such as this one, will continue to be relied upon to generate evidence to policymakers and employers on the effectiveness of workplace breastfeeding interventions. Fourth, the use of a 24‐h recall method may have overestimated EBF (Roberts et al., 2018; Tylleskär et al., 2011). Additionally, mothers might have over‐reported EBF because of social desirability. Finally, this study evaluated the effect of a package of interventions making it impossible to determine which component of the package was more effective. This is because all women employed on the farm were exposed to workplace support policies, all women with children younger than 1 year participated in mother support groups, 91% of women accessed home‐based breastfeeding support by peer educators/community health volunteers, and only a few women (exact number not available) did not utilize the daycare centres because of limited space and personal preferences. Nonetheless, challenges of breastfeeding in the workplace are multifaceted and require a multifaceted approach. Moreover, promotion of breastfeeding is likely to be more effective if delivered concurrently in a combination of settings, including home and community (Sinha et al., 2015).

In conclusion, the baby‐friendly workplace support intervention promoted EBF in this setting and was especially supportive in increasing EBF likelihood beyond 3 months, which is the age in Kenya beyond which employer support for maternity leave ceases for those working in the formal sector. This indicates the need for policies and programmes at workplaces to support women to combine work with breastfeeding. The recently enacted law in Kenya under the Health Act 2017 (The National Council for Law Reporting, 2017), that mandates employers to provide support for women at work, including providing space and facilities to enable breastfeeding or breast milk expression at work is a great start. This is in line with the International Labour Organization Maternity Protection recommendation No. 191 that recommends the provision of lactation facilities at the workplace [International Labour Organization (ILO), 2000]. As our findings indicate, maintaining EBF while working is more likely when employers provide the support that women need to do so. Implementation of the lactation support law in Kenya is therefore likely to promote EBF among working mothers, thereby improving the health, wellbeing and survival of children, and the health and wellbeing of their mothers.

## CONFLICTS OF INTEREST

The authors declare that they have no conflicts of interest.

## CONTRIBUTIONS

EWK‐M was the principal investigator and provided overall leadership to the study; CW, the joint first author, analysed the data and wrote the first draft of the manuscript; EWK‐M, GM, HPPD, LK, SJ, LAD, BS, JA, RL, PG, DJ and FB conceptualized and designed the study; TNM, EWK, PMG, TZ and HPPD supervised and coordinated data collection and intervention implementation; all the authors interpreted the data, reviewed subsequent drafts of the manuscript for important intellectual content and approved the final manuscript for submission.

## TRIAL REGISTRATION

ISRCTN 64692465 (Registered on 21 December 2016).

## Supporting information

**Table S1:** A comparison of the characteristics of mothers and children with complete data and those with missing data at baseline
**Table S2**: Effect of the baby‐friendly workplace support intervention on exclusive breastfeeding after multiple imputation to account for missing data
**Table S3**: Effect of the baby‐friendly workplace support intervention on exclusive breastfeeding stratified by mother's employment status after multiple imputation to account for missing data
**Figure S1: Classification of spheres to target behavioral interventions in Maternal, Newborn and Child Health and Nutrition.** MoH: Ministry of Health; MoLE: Ministry of Labor and Employment; KEPSA: Kenya Private Sector Alliance; CHEW: Community Health Extension Worker; CHV: Community Health Volunteer; MNCH: Maternal, Neonatal and Child Health; BF: Breastfeeding

## Data Availability

The data that support the findings of this study are available on request from the African Population and Health Research Center through the Microdata portal (https://aphrc.org/microdata‐portal/).
