# Supplementary material for: Effect of a baby‐friendly workplace support intervention on exclusive breastfeeding in Kenya
Source: Matern Child Nutr. 2021 Apr 8;17(4):e13191. doi: 10.1111/mcn.13191 (PMC8476432; doi:10.1111/mcn.13191)
Supplement: Supplementary file 1 — Table S1: A comparison of the characteristics of mothers and children with complete data and those with missing data at baseline Table S2: Effect of the baby‐friendly workplace support intervention on exclusive breastfeeding after multiple imputation to account for missing data Table S3: Effect of the baby‐friendly workplace support intervention on exclusive breastfeeding stratified by mother's employment status after multiple imputation to account for missing data Figure S1: Classification of spheres to target behavioral interventions in Maternal, Newborn and Child Health and Nutrition. MoH: Ministry of Health; MoLE: Ministry of Labor and Employment; KEPSA: Kenya Private Sector Alliance; CHEW: Community Health Extension Worker; CHV: Community Health Volunteer; MNCH: Maternal, Neonatal and Child Health; BF: Breastfeeding [file MCN-17-e13191-s001.docx]

**Effect of a baby-friendly workplace support interventions on exclusive breastfeeding in Kenya**

**Supporting Information for review and publication**

Supplemental Table 1: A comparison of the characteristics of mothers and children with complete data and those with missing data at baseline

| **Characteristics** | | **Complete data (N = 223)** | **Missing data (N = 47)** | **P value** |
| --- | --- | --- | --- | --- |
| **Child’s characteristics** | |  |  |  |
| Age, months | |  |  | 0.857 |
|  | 0.0 – 2.9 | 199 (53.9) | 26 (55.3) |  |
|  | 3.0 – 5.9 | 170 (46.1) | 21 (44.7) |  |
| Sex | |  |  | 0.940 |
|  | Male | 202 (54.7) | 26 (55.3) |  |
|  | Female | 167 (45.3) | 21 (44.7) |  |
| **Mother’s characteristics** | |  |  |  |
| Age, years, mean ± SD | | 26.4 ± 6.4 | 27.3 ± 6.3 | 0.360^†^ |
| Parity | |  |  | 0.214 |
|  | 1 | 101 (27.4) | 8 (17.0) |  |
|  | 2 | 82 (22.2) | 16 (34.0) |  |
|  | 3 | 78 (21.1) | 11 (23.4) |  |
|  | 4+ | 108 (29.3) | 12 (25.5) |  |
| Ethnic group | |  |  | 0.970 |
|  | Kalenjin | 187 (50.7) | 23 (48.9) |  |
|  | Kisii | 93 (25.2) | 12 (25.5) |  |
|  | Other | 89 (24.1) | 12 (25.5) |  |
| Education | |  |  | 0.169 |
|  | Primary or less | 189 (51.2) | 18 (38.3) |  |
|  | Secondary | 131 (35.5) | 19 (40.4) |  |
|  | Tertiary | 49 (13.3) | 10 (21.3) |  |
| Religion | |  |  | 0.075^‡^ |
|  | Christian | 359 (97.3) | 42 (89.4) |  |
|  | Other | 10 (2.7) | 5 (10.6) |  |
| Marital status | |  |  | 0.010^‡^ |
|  | Not in a union | 77 (20.9) | 4 (8.5) |  |
|  | In a union | 292 (79.1) | 43 (91.5) |  |
| Employment status | |  |  | 0.660^‡^ |
|  | Employed in agricultural estate | 126 (34.2) | 18 (38.3) |  |
|  | Employed elsewhere | 18 (4.9) | 4 (8.5) |  |
|  | Unemployed | 225 (61.0) | 25 (53.2) |  |

Data are presented as n (%) except for mother’s age which is presented as mean ± SD. P values are from Pearson’s Chi-squared test unless specified otherwise.

^†^Independent samples t-test

^‡^Fisher's exact test

Supplemental Table 2: Effect of the baby-friendly workplace support intervention on exclusive breastfeeding after multiple imputation to account for missing data

| Study group | Exclusively breastfed | Unadjusted analysis | Propensity score weighted analysis | Multivariable adjusted analysis^†^ |
| --- | --- | --- | --- | --- |
|  | % | RR (95% CI) | RR (95% CI) | RR (95% CI) |
| **0.0-5.9 months** |  |  |  |  |
| Non-treated (N = 270) | 20.6 | 1 | 1 | 1 |
| Treated (N = 146) | 80.8 | 3.94 (2.98, 5.21) | 3.84 (2.89, 5.10) | 3.75 (2.81 (5.00) |
| **0.0-2.9 months** |  |  |  |  |
| Non-treated (N = 142) | 31.0 | 1 | 1 | 1 |
| Treated (N = 83) | 88.0 | 2.84 (2.12, 3.82) | 2.79 (2.07, 3.76) | 2.70 (1.99, 3.66) |
| **3.0-5.9 months** |  |  |  |  |
| Non-treated (N = 128) | 9.0 | 1 | 1 | 1 |
| Treated (N = 63) | 71.4 | 7.99 (4.34, 14.71) | 7.66 (4.09, 14.37) | 7.75 (4.22, 14.24) |

^†^adjusted for child’s sex, mother’s age; religion, marital status and employment status

Supplemental Table 3: Effect of the baby-friendly workplace support intervention on exclusive breastfeeding stratified by mother’s employment status after multiple imputation to account for missing data

| Study group | Mother employed in the agricultural estate (N=144) | | |  | Mother unemployed (N=250) | | |
| --- | --- | --- | --- | --- | --- | --- | --- |
|  | % exclusively breastfed | Propensity score weighted analysis | Multivariable adjusted analysis^†^ |  | % exclusively breastfed | Propensity score weighted analysis | Multivariable adjusted analysis^†^ |
|  |  | RR (95% CI) | RR (95% CI) |  |  | RR (95% CI) | RR (95% CI) |
| Non-treated | 21.6 | 1 | 1 |  | 20.7 | 1 | 1 |
| Treated | 83.3 | 3.84 (2.40–6.14) | 3.74 (2.37–5.91) |  | 78.7 | 3.66 (2.54–5.28) | 3.50 (2.41–5.06) |

^†^adjusted for child’s sex, mother’s age; religion, and marital status


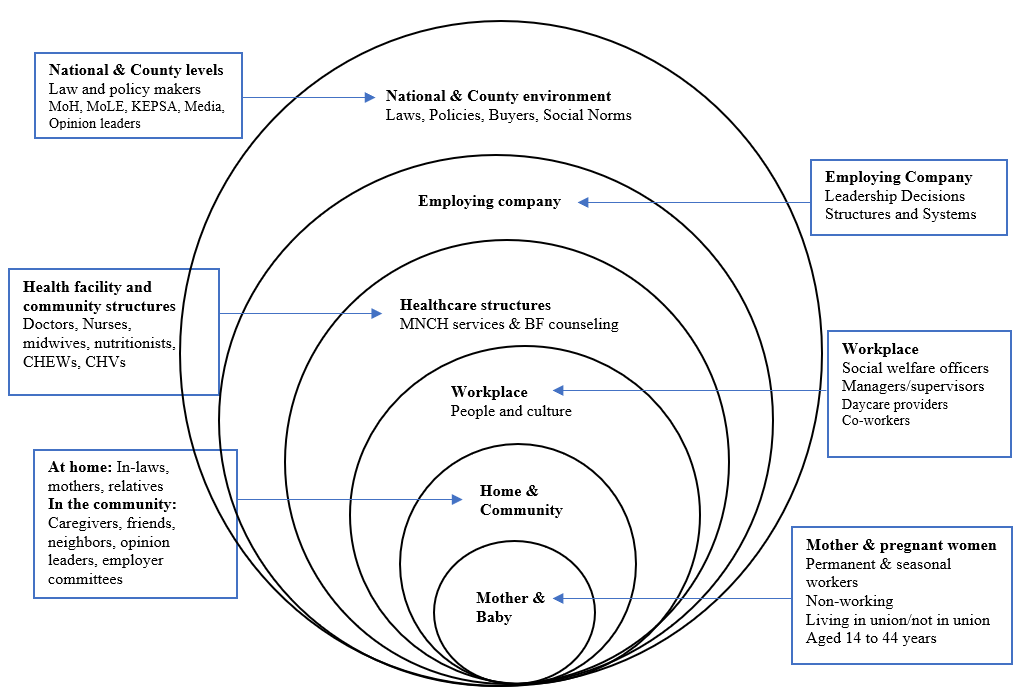


**Supplemental Figure 1: Classification of spheres to target behavioral interventions in Maternal, Newborn and Child Health and Nutrition.** MoH: Ministry of Health; MoLE: Ministry of Labor and Employment; KEPSA: Kenya Private Sector Alliance; CHEW: Community Health Extension Worker; CHV: Community Health Volunteer; MNCH: Maternal, Neonatal and Child Health; BF: Breastfeeding
